# Supplementary material for: Architecture of the centriole cartwheel‐containing region revealed by cryo‐electron tomography
Source: EMBO J. 2020 Sep 20;39(22):e106246. doi: 10.15252/embj.2020106246 (PMC7667884; doi:10.15252/embj.2020106246)
Supplement: Supplementary file 1 — Appendix [file EMBJ-39-e106246-s001.pdf]

Appendix

Architecture of the centriole cartwheel-containing region revealed by cryo-electron tomography

Nikolai Klena[†], Maeva Le Guennec[†], Anne-Marie Tassin, Hugo van den Hoek, Philipp S. Erdmann, Miroslava Schaffer, Stefan Geimer, Gabriel Aeschlimann, Lubomir Kovacik, Yashar Sadian, Kenneth N. Goldie, Henning Stahlberg, Benjamin D. Engel, Virginie Hamel and Paul Guichard

*Correspondence to: ben.engel@helmholtz-muenchen.de, virginie.hamel@unige.ch and paul.guichard@unige.ch

Table of Contents

Appendix Figure S1 - page 2
Appendix Figure S2 - pages 3-4
Appendix Figure S3 – page 5
Appendix Figure S4- page 6
Appendix Figure S5 – page 7-8
Appendix Figure S6 – page 9
Appendix Figure S7- page 10
Appendix Figure S8 – page 11
Appendix Figure S9 – page 12

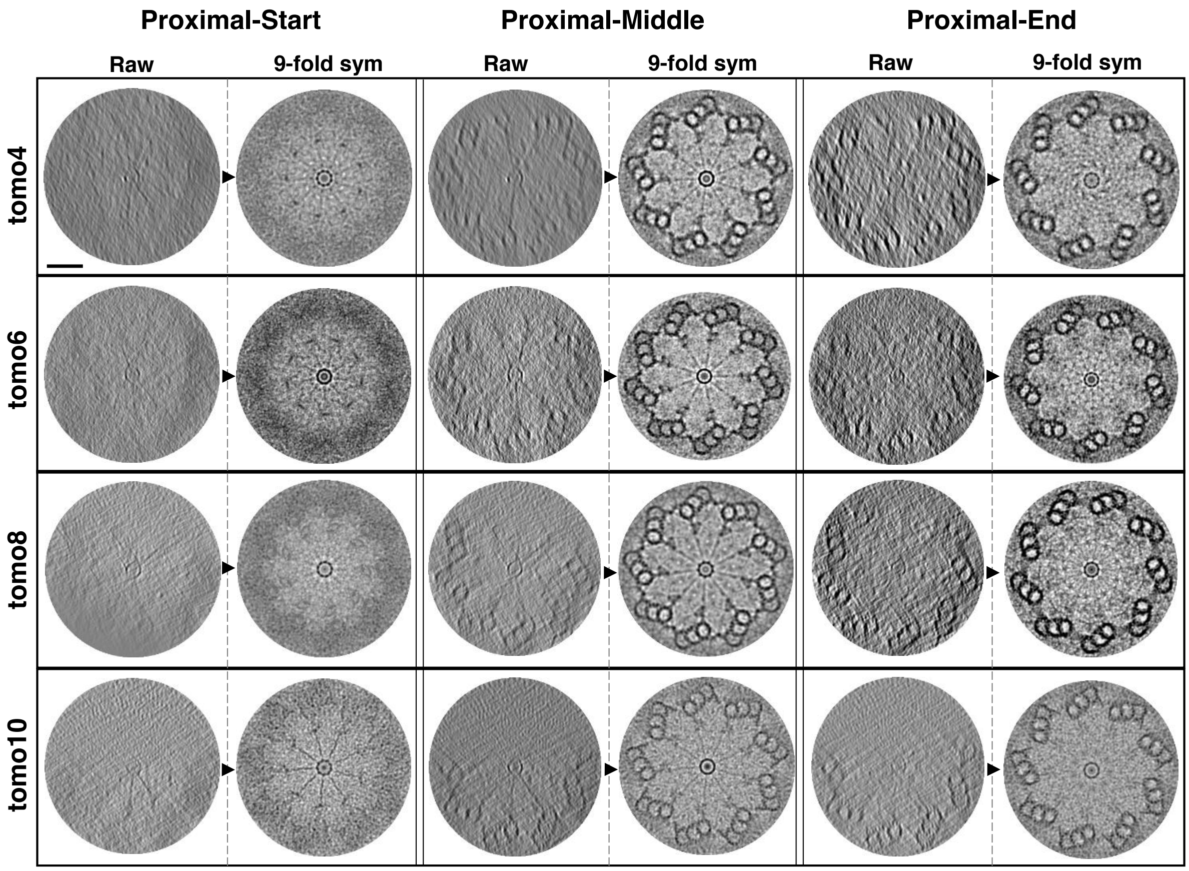

Appendix Figure S1. The cartwheel-containing region in mature *C. reinhardtii* centrioles.

From four tomograms, three regions were extracted every 35 nm along the proximal region corresponding to the Proximal-Start, Proximal-Middle, and Proximal-End regions. Each image corresponds to a projection of about 27 nm. To improve visualization, we applied a nine-fold symmetrization of the image (displayed to the right of the raw image, separated by a dashed grey line and a black arrowhead). Scale bar, 100 nm.

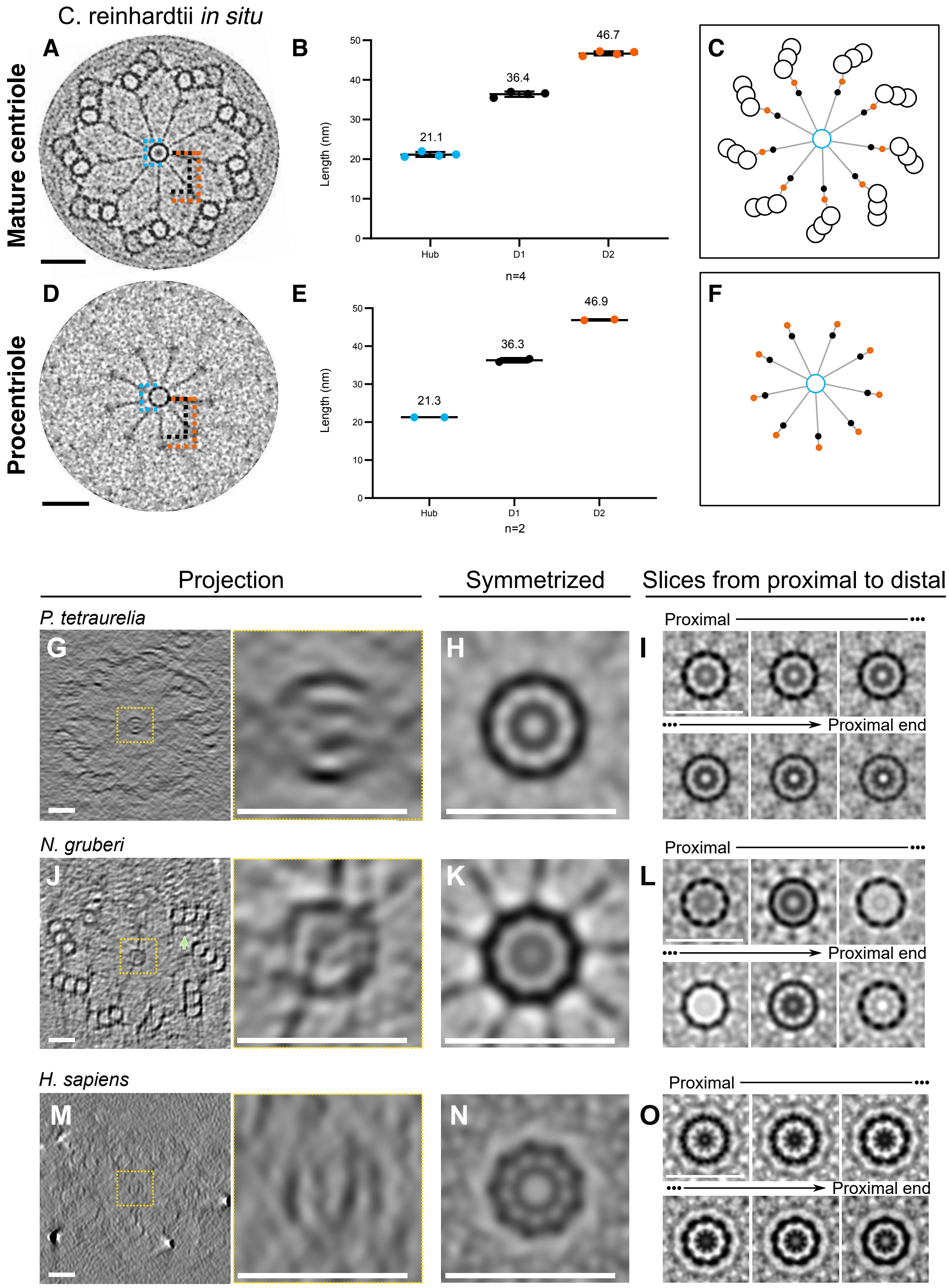

Appendix Figure S2. *In situ* spoke architecture of the *C. reinhardtii* cartwheel, and conservation of the cartwheel inner densities.

(A) Nine-fold symmetrized cross section of cartwheel-containing region from a *in situ* tomogram of a *C. reinhardtii* mature centriole, The dashed blue line indicates central hub diameter. Dashed black and dark orange lines indicate distances from the external edge the central hub to D1 and D2 densities of the radial spoke, respectively.

(B) Measurements of cartwheel features: mean diameter of the central hub (blue), distance from the central hub to D1 (black) and D2 (dark orange) densities in mature centrioles (n = 4). Mean values are displayed above the data range.
(C) Model of cartwheel organization and distance from the central hub to D1 and D2 in mature centrioles. Central hub, blue; spoke, grey; D1, black circle; D2, dark orange circle.
(D) Nine-fold symmetrized cross section of cartwheel-containing region from *in situ* tomogram of a *C. reinhardtii* procentriole. The dashed blue line indicates central hub diameter. Dashed black and dark orange lines indicate distances from the external edge the central hub to D1 and D2 densities of the radial spoke, respectively.
(E) Measurements of cartwheel features: mean diameter of the central hub (blue), distance from the central hub to D1 (black) and D2 (dark orange) densities in procentrioles (n = 2). Mean values are displayed above the data range.
(F) Model of cartwheel organization and distance from the central hub to D1 and D2 in procentrioles. Central hub, blue; spoke, grey; D1, black circle; D2, dark orange circle.
(G) Cryo-electron tomogram cross section depicting top view of the proximal region of *P. tetraurelia*. Yellow dashed box indicates central hub with the corresponding zoom on the right. Scale bar, 40 nm.
(H) Nine-fold symmetrized image corresponding to the panel in G. Scale bar, 40 nm.
(I) Symmetrized 4 nm serial projections through one central hub of *P. tetraurelia*. Scale bar, 40 nm.
(J) Cryo-electron tomogram cross-section depicting top view of the proximal region of *N. gruberi*. Yellow dashed box indicates central hub with the corresponding zoom on the right. Scale bar, 40 nm.
(K) Nine-fold symmetrized image corresponding to the panel in J. Scale bar: 40 nm.
(L) Symmetrized 4 nm serial projections through one central hub of *N. gruberi*. Scale bar, 40 nm.
(M) Cryo-electron tomogram cross-section depicting top view of the proximal region of *H. sapiens* (M). Yellow dashed box indicates central hub with the corresponding zoom on the right. Scale bar, 40 nm.
(N) Nine-fold symmetrized image corresponding to the panel in M. Scale bar, 40 nm.
(O) Symmetrized 4 nm serial projections through one central hub of *H. sapiens*. Scale bar, 40 nm.

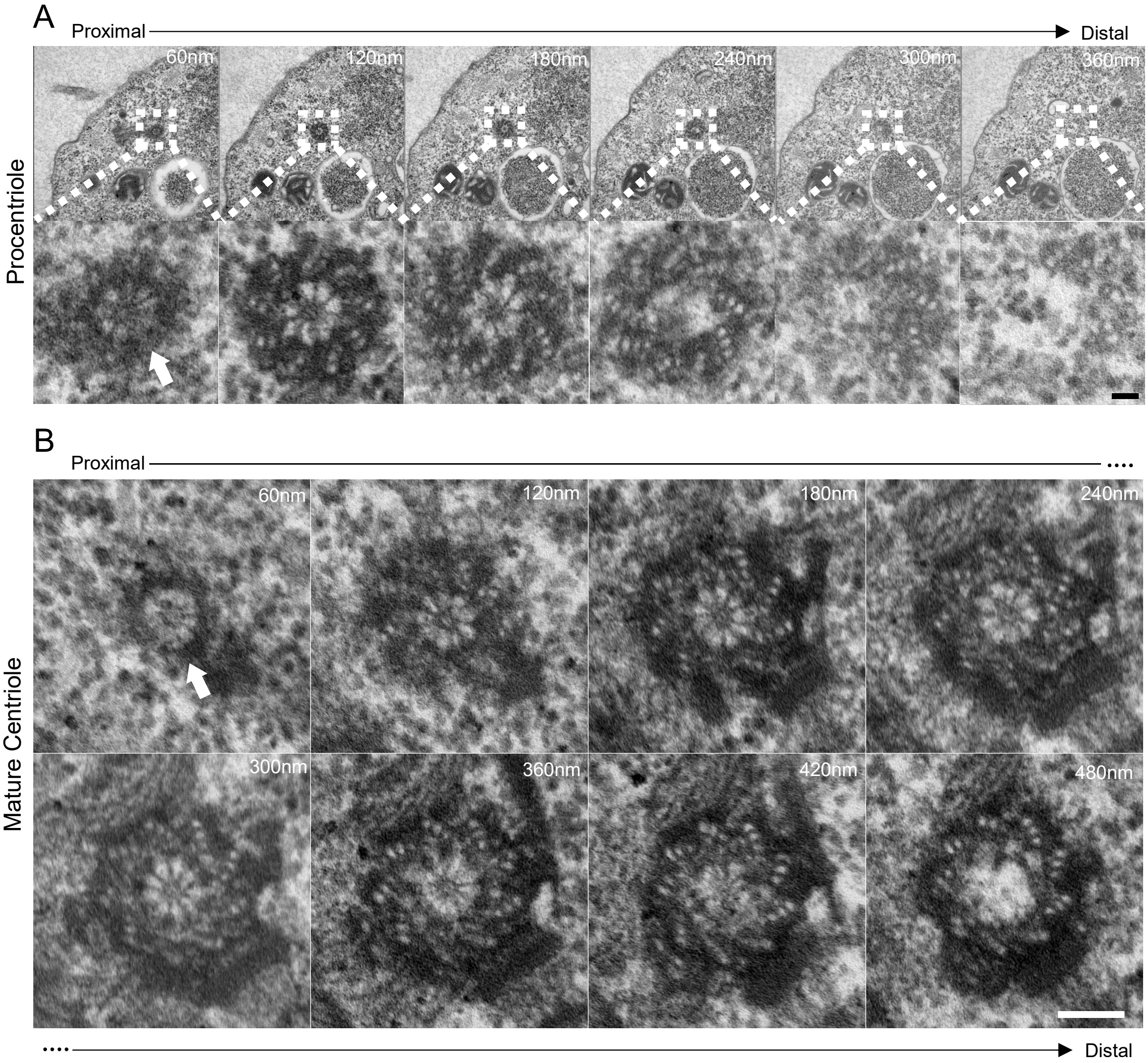

Appendix Figure S3. Resin-embedded *N. gruberi* cells display the proximal cartwheel protrusion in both mature centrioles and procentrioles.

(A) Serial sections through a procentriole in a resin-embedded *N. gruberi* cell, moving from proximal (left) to distal (right). White-dashed box denotes the zoomed region in the bottom panel. White arrow denotes the cartwheel extending beyond the proximal microtubule triplet region. Scale bar, 50nm.

(B) Serial sections through a mature centriole in a resin-embedded *N. gruberi* cell, moving from proximal (left) to more distal (right). White arrow denotes the cartwheel extending beyond the proximal microtubule triplet region. Scale bar, 100nm

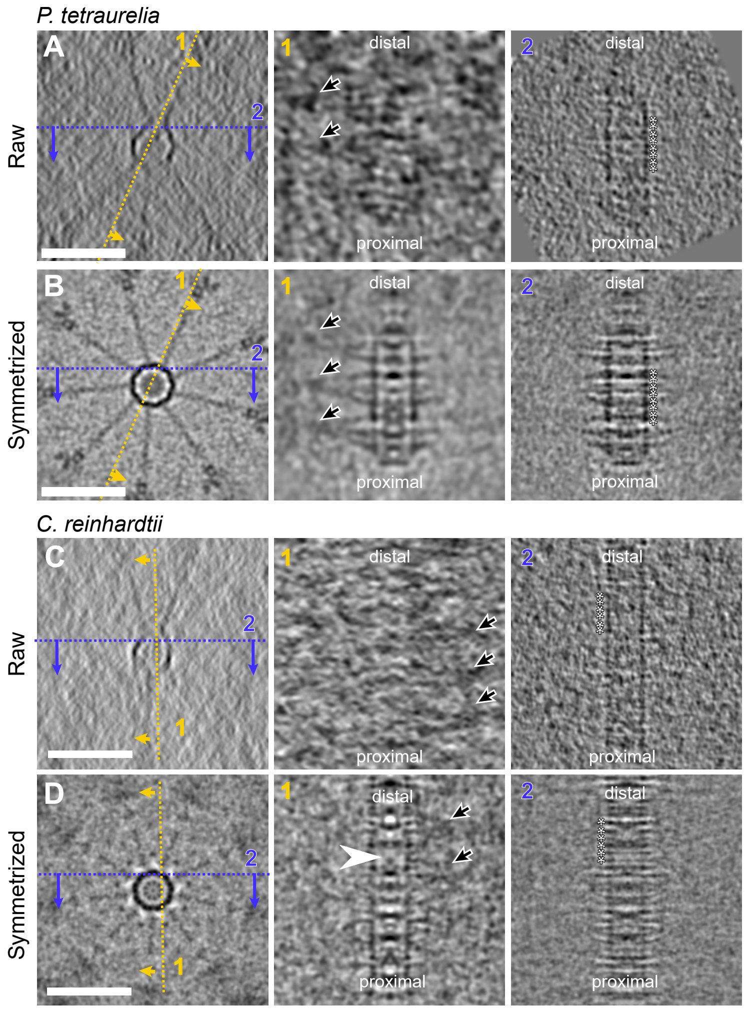

Appendix Figure S4. Raw and symmetrized cartwheels from *P. tetraurelia* and *C. reinhardtii*.

(A) Cryo-electron tomogram sections displaying the cartwheels of *P. tetraurelia* from top view (left panels) and side views (middle panel focusing on the spokes and right panel on the central hub).

(B) Corresponding nine-fold symmetrized image displaying the cartwheels *of P. tetraurelia* from top view (left panels) and side views (middle and right panels).

(C) Cryo-electron tomogram sections displaying the cartwheels of *C. reinhardtii* from top view (left panels) and side views (middle panel focusing on the spokes and right panel on the central hub).

(D) Corresponding nine-fold symmetrized image displaying the cartwheels *of C. reinhardtii* from top view (left panels) and side views (middle and right panels).

Data information: Scale bars: 50 nm. Dashed yellow lines and arrows indicate the position and direction of the reslice to visualize the radial spokes (1), Dashed blue line and arrows indicate the position and direction of the reslice to visualize the central hub (2). Black arrows indicate the position of merged spokes corresponding to the D1 density (1). White asterisks denote positions of central hub ring subunits (2). Note that all images have been smoothed using a 3D Gaussian filter to increase the contrast.

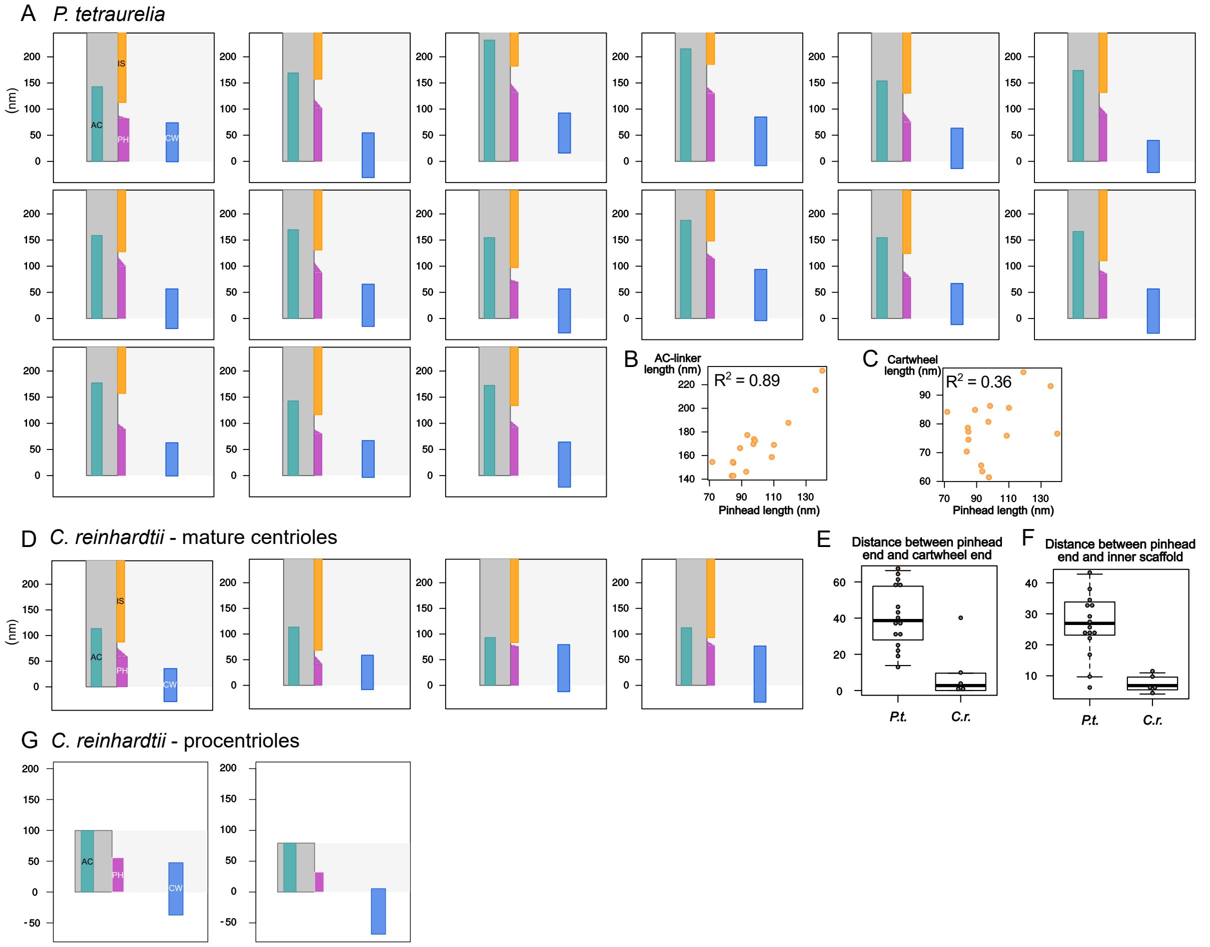

Appendix Figure S5. Boundaries of the proximal region's structural features in *P. tetraurelia* and *C. reinhardtii* centrioles.

(A) Positions of the different structures along the proximal to distal axis of 15 different *P. tetraurelia* centrioles. Dark blue, cartwheel (CW); purple, pinhead (PH); turquoise, A-C linker (AC); orange, inner scaffold (IS); dark grey, microtubule wall.

(B) Correlation plot depicting A-C linker length versus pinhead length from *P. tetraurelia* centrioles. N = 16, Pearson correlation coefficient 0.89.

(C) Correlation plot depicting cartwheel length versus pinhead length from *P. tetraurelia* centrioles. N = 16, Pearson correlation coefficient 0.36.

(D) Positions of the different structures along the proximal to distal axis of 4 different *C. reinhardtii* mature centrioles. Same color code as panel A.

(E) Distance between the end of the pinhead region and the end of the cartwheel region. (n = 16, *P. tetraurelia*; n = 5, *C. reinhardtii*). Black thick line is the median, box edges are the lower and upper quartiles and whiskers segment correspond to the lowest and highest points (excluding outliers, that are points outside the box length *1.5)

(F) Distance between the end of the pinhead region and the beginning of the inner scaffold region (n = 15, *P. tetraurelia*; n = 5, *C. reinhardtii*). Black thick line is the median, box edges are the lower and upper quartiles and whiskers segment correspond to the lowest and highest points (excluding outliers, that are points outside the box length *1.5).

(G) Positions of the different structures along the proximal to distal axis of 2 different *C. reinhardtii* procentrioles.

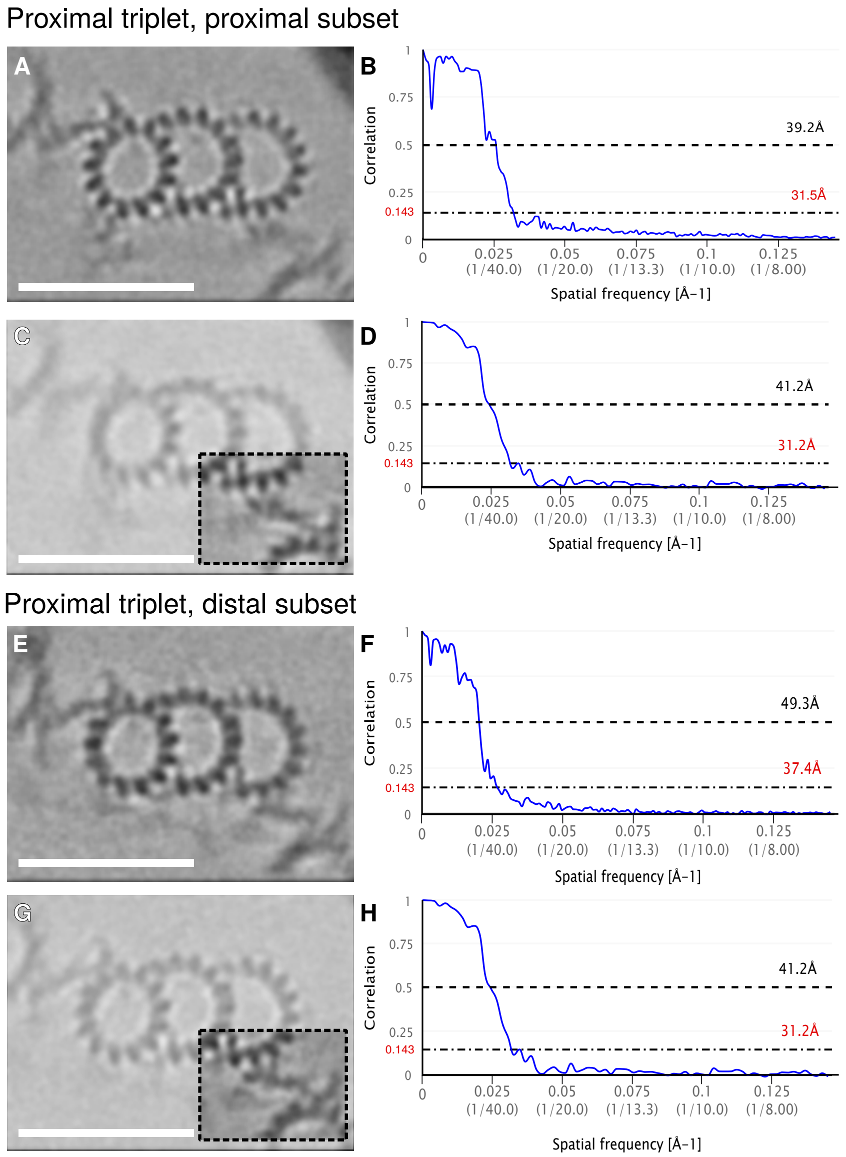

Appendix Figure S6. Resolution of the subtomogram averages generated from the *P. tetraurelia* proximal centriole.

(A-H) Z-projections of the obtained 3D maps (panels A, C, E, G) and their corresponding resolutions estimation by FSC curve (panels B, D, F, H). Initial maps were obtained by averaging the entire microtubule triplet from only the most proximal region (A) or the more distal part of the proximal region (E). Additional maps were made by local refinement of the A-C linker (dashed squared area) (C, G). Scale bars, 50 nm.

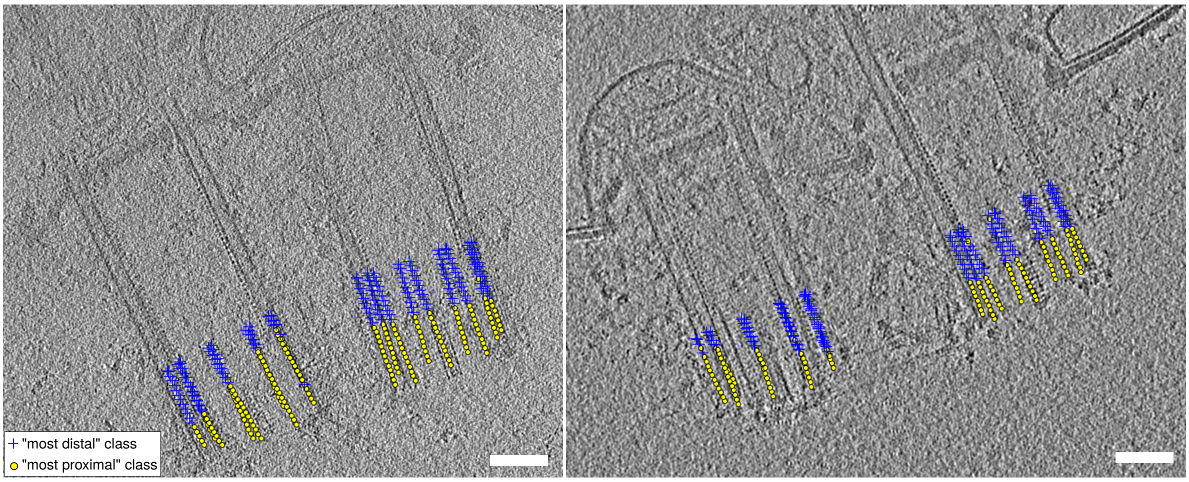

Appendix Figure S7. Distribution of subtomograms from the proximal microtubule triplet.

Examples of subtomogram positions after classification in *P. tetraurelia* centrioles. Yellow dots represent subtomograms classified as "most proximal". Blue crosses represent subtomograms classified as "most distal". Scale bars, 100 nm.

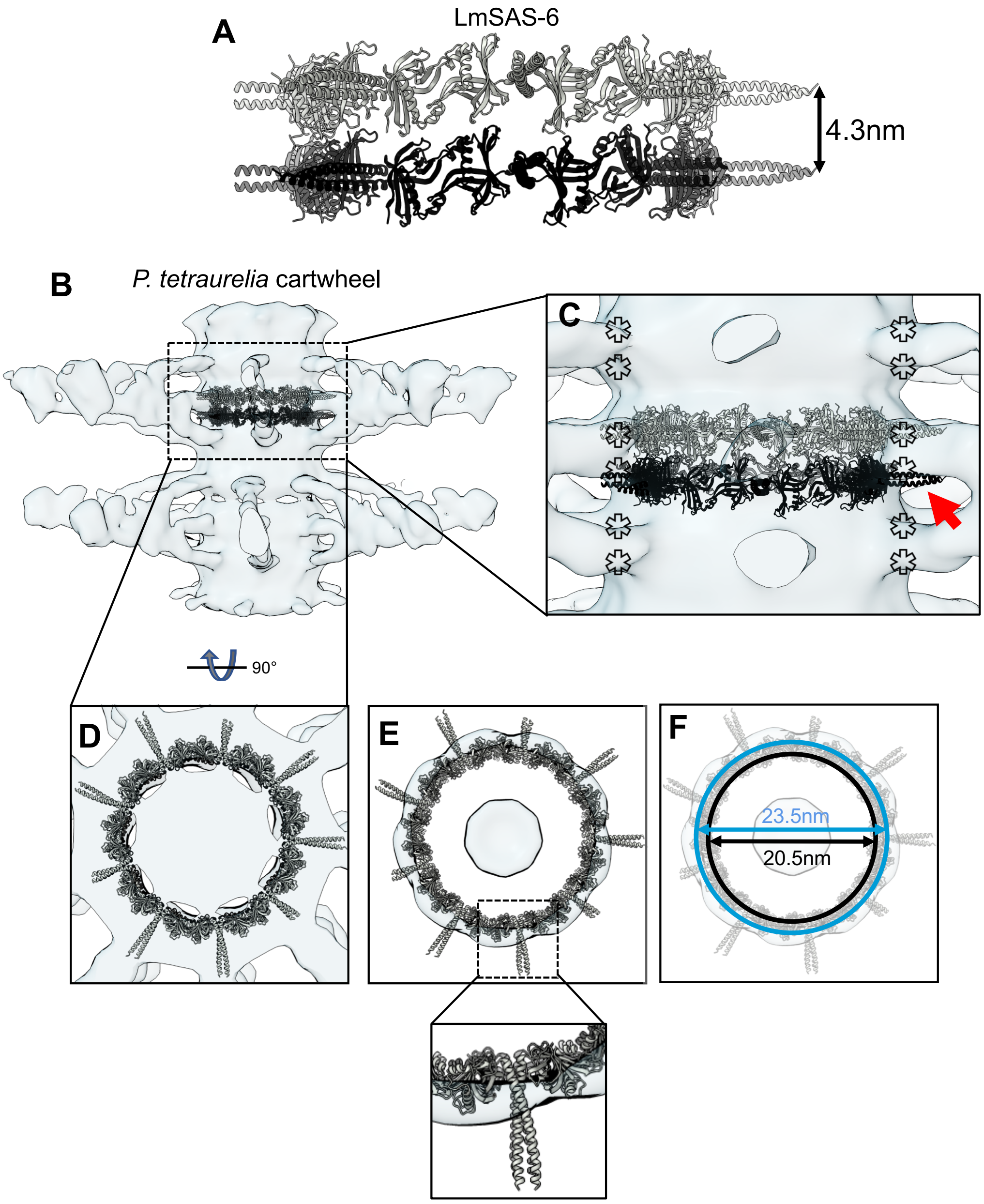

Appendix Figure S8. Fitting LmSAS-6 ring into *P. tetraurelia* cartwheel map
(A) Crystal structures of 2 *Leishmania* SAS-6 rings (pdbID 4ckp), placed in register and distanced by 4.3 nm.
(B) LmSAS-6 rings fitted into the *P.tetraurelia* cryo-ET map using ChimeraX.
(C) Zoomed inset highlighting that one coiled coil of LmSAS-6 ring-pair model doesn't fit inside the emanating spoke cryo-ET density (red arrow). Asterisks indicate the position of the ring-pairs densities in the cryoET map.
(D-F) Zoomed insets of the top view of LmSAS-6 rings fitted into the *P.tetraurelia* cryo-ET map at high (D) or low (E) threshold. (F) Diameter comparison between the LmSAS-6 ring and the hub diameter of the *P. tetraurelia* cartwheel. The difference in diameter does not allow LmSAS-6 to be fitted correctly in the 3D map.

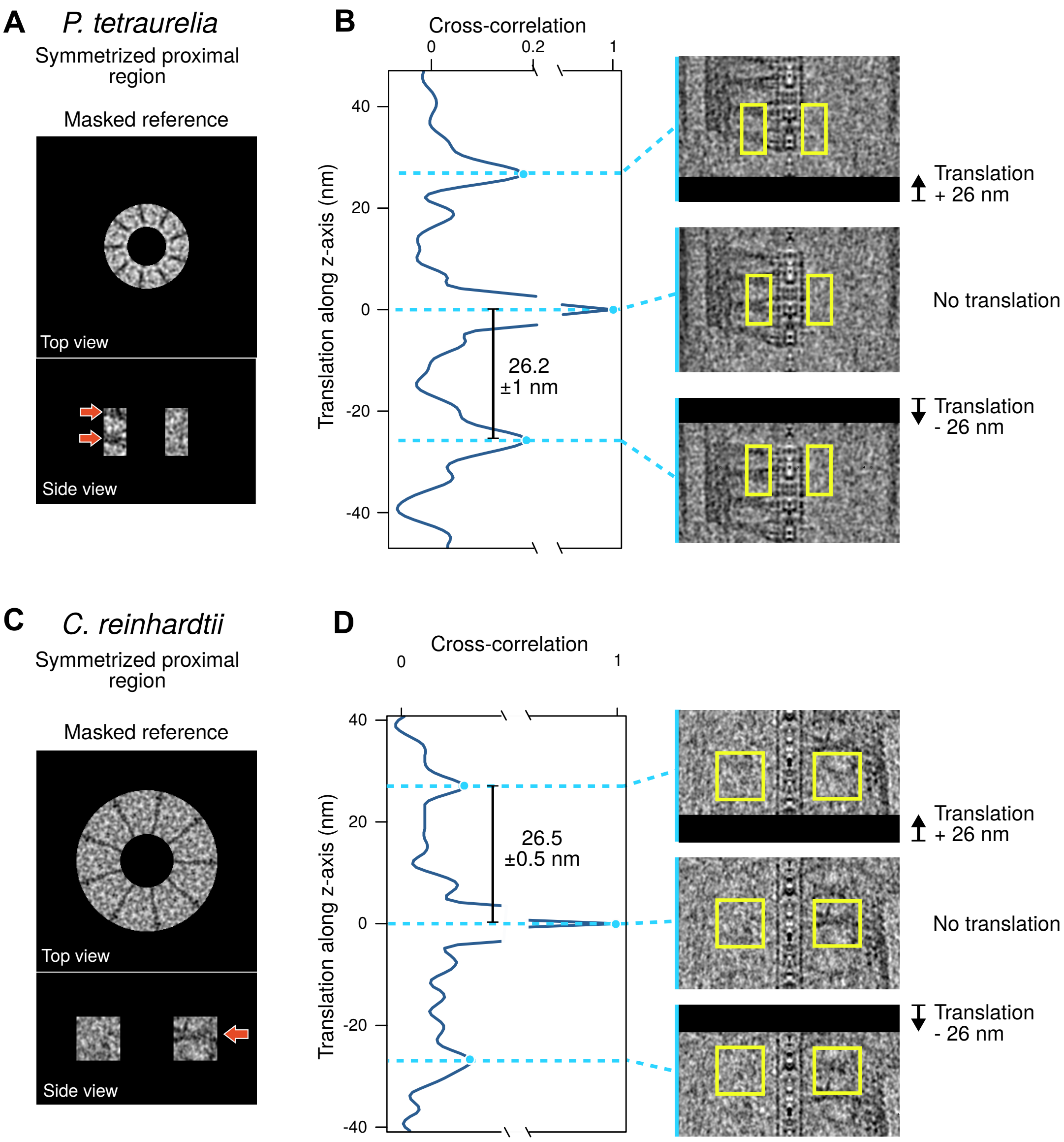

Appendix Figure S9. Translational analysis along the cartwheel length.
(A) Top and side views of symmetrized proximal region from *P. tetraurelia* centriole masked around the cartwheel spokes (orange arrows) for the translational analysis.
(B) Cross-correlation between the translated volume and the masked reference. The right panel illustrates the position of the translated volume at each peak. The yellow squares indicate where the cross-correlation is computed. See more details in material and methods.
(C) Top and side views of symmetrized proximal region from *C. reinhardtii* centriole masked around the cartwheel spokes (orange arrows) for the translational analysis.
(D) Cross-correlation between the translated volume and the masked reference. The right panel illustrates the position of the translated volume at each peak. The yellow squares indicate where the cross-correlation is computed. See more details in material and methods.
