# Supplementary material for: Architecture of the centriole cartwheel‐containing region revealed by cryo‐electron tomography
Source: EMBO J. 2020 Sep 20;39(22):e106246. doi: 10.15252/embj.2020106246 (PMC7667884; doi:10.15252/embj.2020106246)
Supplement: Supplementary file 2 — Expanded View Figures PDF [file EMBJ-39-e106246-s002.pdf]

Expanded View Figures

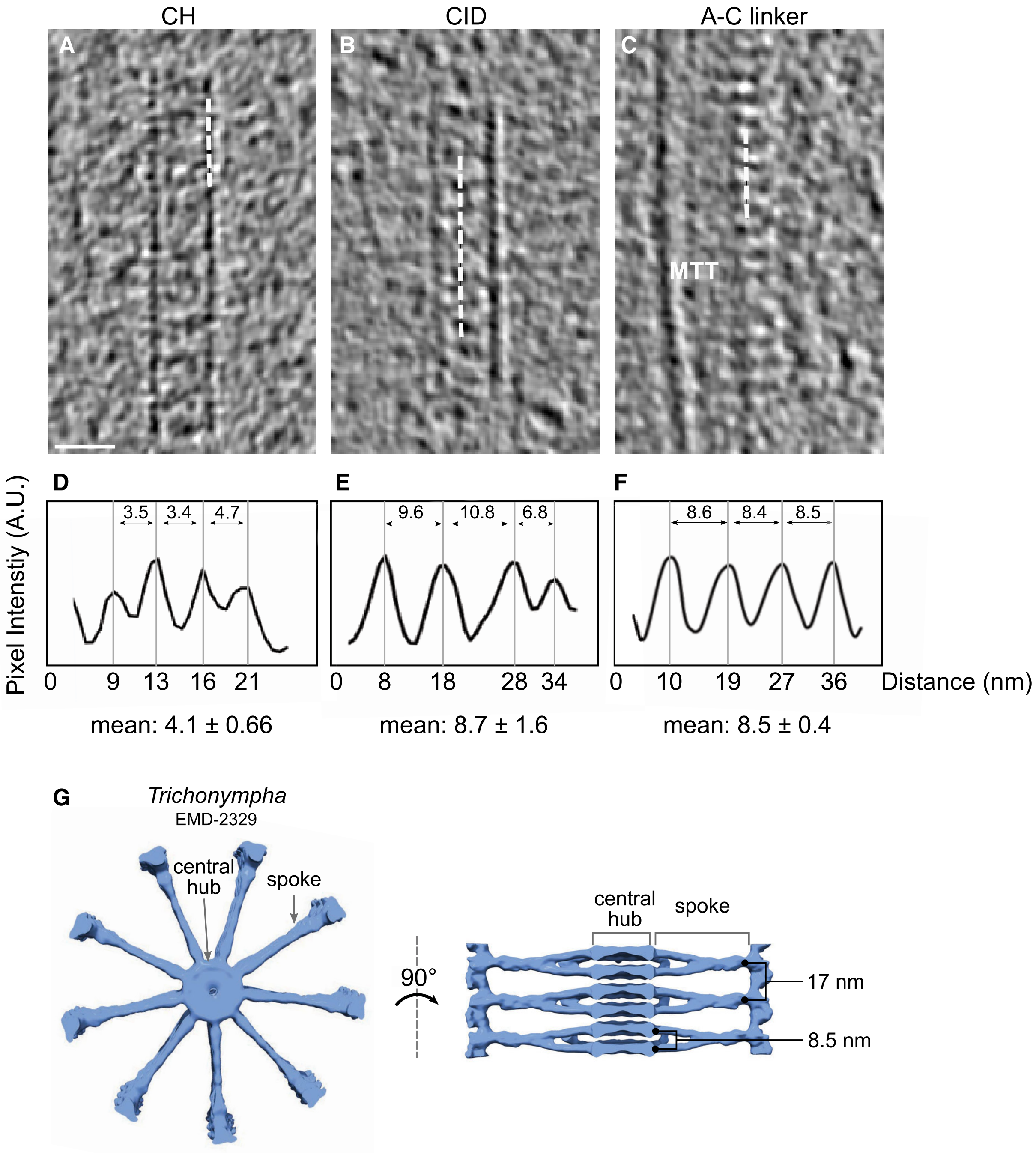

Figure EV1. Periodicity along the central hub, cartwheel inner densities, and A-C linker in *C. reinhardtii in situ* centrioles.

A–C Cryo-ET sections depicting representative central hub (CH) (A), several cartwheel inner densities (CIDs) (B), and A-C linker (C). Dashed white line denotes region from which plot profiles were generated. Microtubule triplet, MTT. Scale bar, 25 nm.

D–F Plot profiles with their associated mean periodicity displayed below.

G Top and side views of *Trichonympha* cartwheel and associated periodicities from (Guichard *et al*, 2013).

 © 2020 The Authors

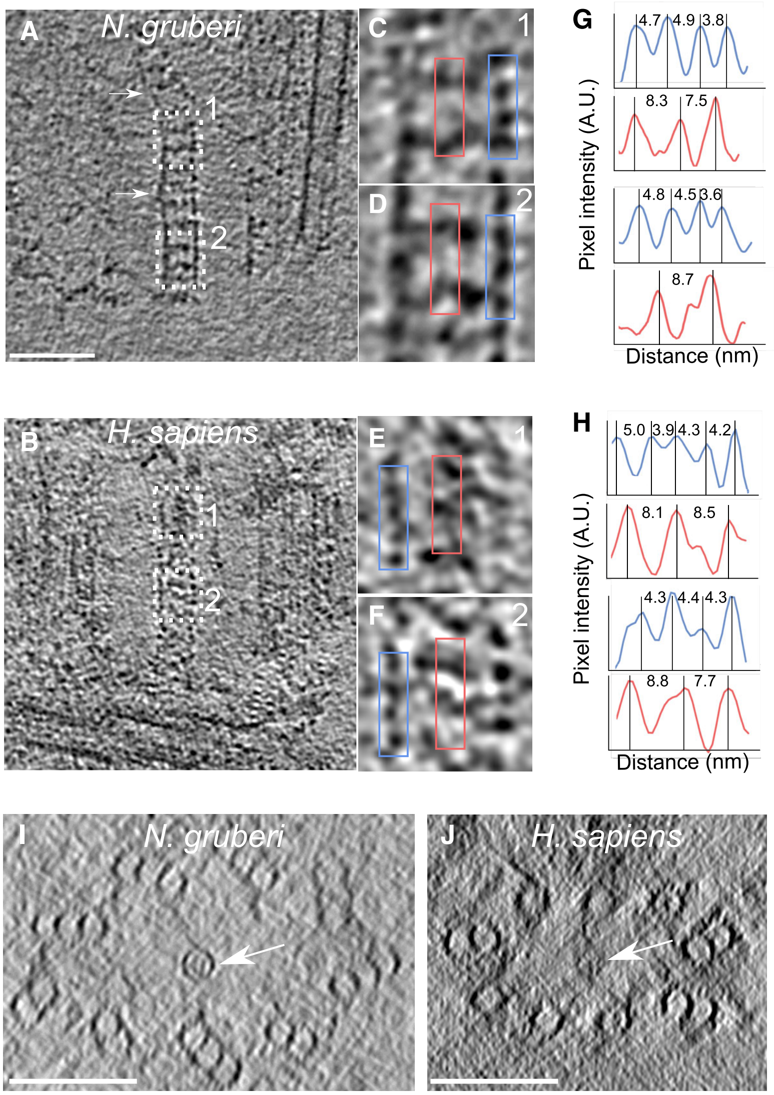

Figure EV2. Additional examples of the cartwheel periodicities in *N. gruberi* and *H. sapiens*.

A–F Side views of cartwheels in *N. gruberi* (A) and *H. sapiens* (B) with corresponding insets (C-F) highlighting the hub periodicities (blue) and CIDs (red). Scale bar, 50 nm.

G, H Plot profiles of the boxed regions in (C-F) depicting an average periodicity of the hub (blue) of 4.4 nm in *N. gruberi* and 4.2 nm in *H. sapiens* as well as for the CIDs with an average periodicity of 8.3 nm in *N. gruberi* and 8.1 nm in *H. sapiens* (see Fig 2).

I, J Top views of representative, compressed *N. gruberi* (I) and *H. sapiens* (J) centrioles displaying that the central hub of the cartwheel is off-centered (white arrows). Scale bars, 100 nm.

© 2020 The Authors

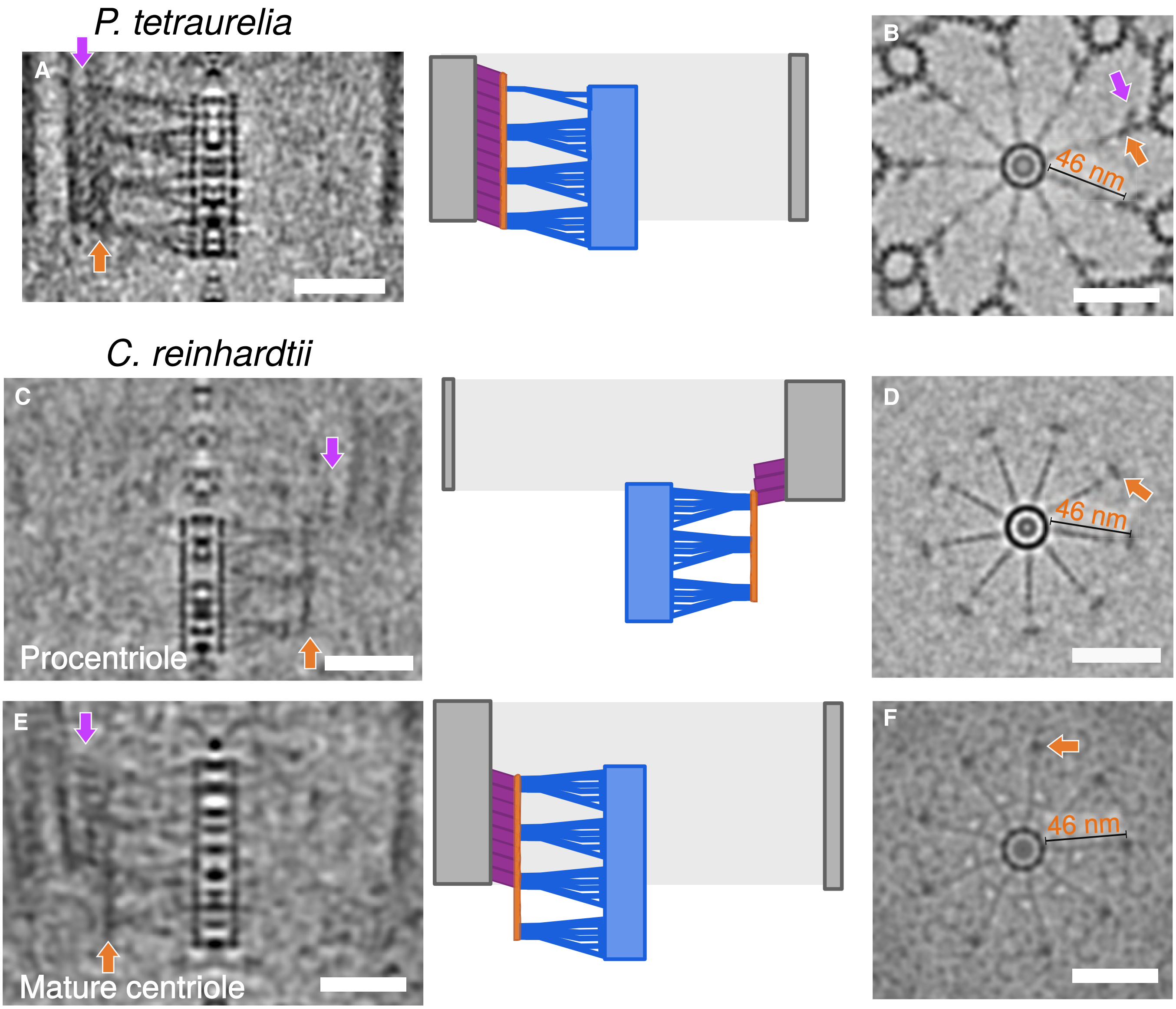

Figure EV3. Native architecture of the proximal cartwheel extension.

A Side view of a symmetrized proximal region (left panel) from *P. tetraurelia* with the corresponding schematic representation (right panel) highlighting the different structural elements: cartwheel, blue; D2-rod, dark orange; pinhead, purple; MTT, gray. Scale bar, 50 nm.

B Top view of symmetrized proximal region from *P. tetraurelia*. The purple arrow marks the pinhead position. Dark orange arrow indicates the D2-rod positioned 46 nm away from the central hub. Scale bar, 50 nm.

C Side view of a symmetrized proximal region (left panel) from a *C. reinhardtii* procentriole with the corresponding schematic representation (right panel) highlighting the different structural elements: cartwheel, blue; D2-rod, dark orange; pinhead, purple; MTT, gray. Scale bar, 50 nm.

D Top view of symmetrized proximal region from a *C. reinhardtii* procentriole. For *C. reinhardtii*, the top view was obtained by z-projecting the protruding cartwheel region only. Dark orange arrow indicates the D2-rod positioned 46 nm away from the central hub. Scale bar, 50 nm.

E Side view of a symmetrized proximal region (left panel) from a *C. reinhardtii* mature centriole with the corresponding schematic representation (right panel) highlighting the different structural elements: cartwheel, blue; D2-rod, dark orange; pinhead, purple; MTT, gray. Scale bar, 50 nm.

F Top view of symmetrized proximal regions from a *C. reinhardtii* mature centriole. For *C. reinhardtii*, the top view was obtained by z-projecting the protruding cartwheel region only. Dark orange arrow indicates the D2-rod positioned 46 nm away from the central hub. Scale bar, 50 nm.

© 2020 The Authors

Figure EV4. Cartwheel spoke organization in *P. tetraurelia* and *C. reinhardtii* from the central hub through the pinhead.

A, B Serial z-projections of ~4 nm thickness through subtomogram averages of *P. tetraurelia* (A) and *C. reinhardtii* (B) cartwheels. The left-most z-projections display the central hub, and the right-most projections show the pinhead. Yellow dashed lines delineate one repeat unit of the cartwheel. Blue circles indicate central hub ring pairs, white arrows mark individual spokes, white arrowheads mark fused spokes, white dashed lines indicate the spoke tilt, black arrows with a line mark the final merged spoke (D1 density) longitudinally spaced every 25 nm, and dark orange arrows indicate the D2-rod. Scale bars, 50 nm.

C Three-dimensional rendering of the cartwheel reconstruction from *C. reinhardtii.* Right panel, cartwheel oriented along the proximal–distal axis (left side) and oriented along the inverted proximal–distal axis (right side), showing the slight asymmetry of spoke inclination. Dark orange arrows indicate the D2-rod. Dashed yellow box, inset of one spoke unit (left panel), with the major and minor tilt angles of the spokes relative to the central hub. White asterisks denote subunits of ring pairs.

D Bandpass filter applied to a *P. tetraurelia* subtomogram average projection with a cutoff at 38 Å. Blue arrows denote positions of the associated plot profiles through the central hub for the unfiltered (left) and filtered projections (right), respectively. The unfiltered projection displays a mean periodicity of 4.0 ± 1.3 nm (SEM), while the projection filtered to 38 Å displays a mean periodicity of 8.6 ± 0.4 nm (SEM). Scale bar, 20 nm.

E Plot profile along the previously published *Trichonympha* central hub (dark blue) (EMD-2329) displaying a longitudinal periodicity of 8.2 nm. Scale bar, 20 nm.

© 2020 The Authors

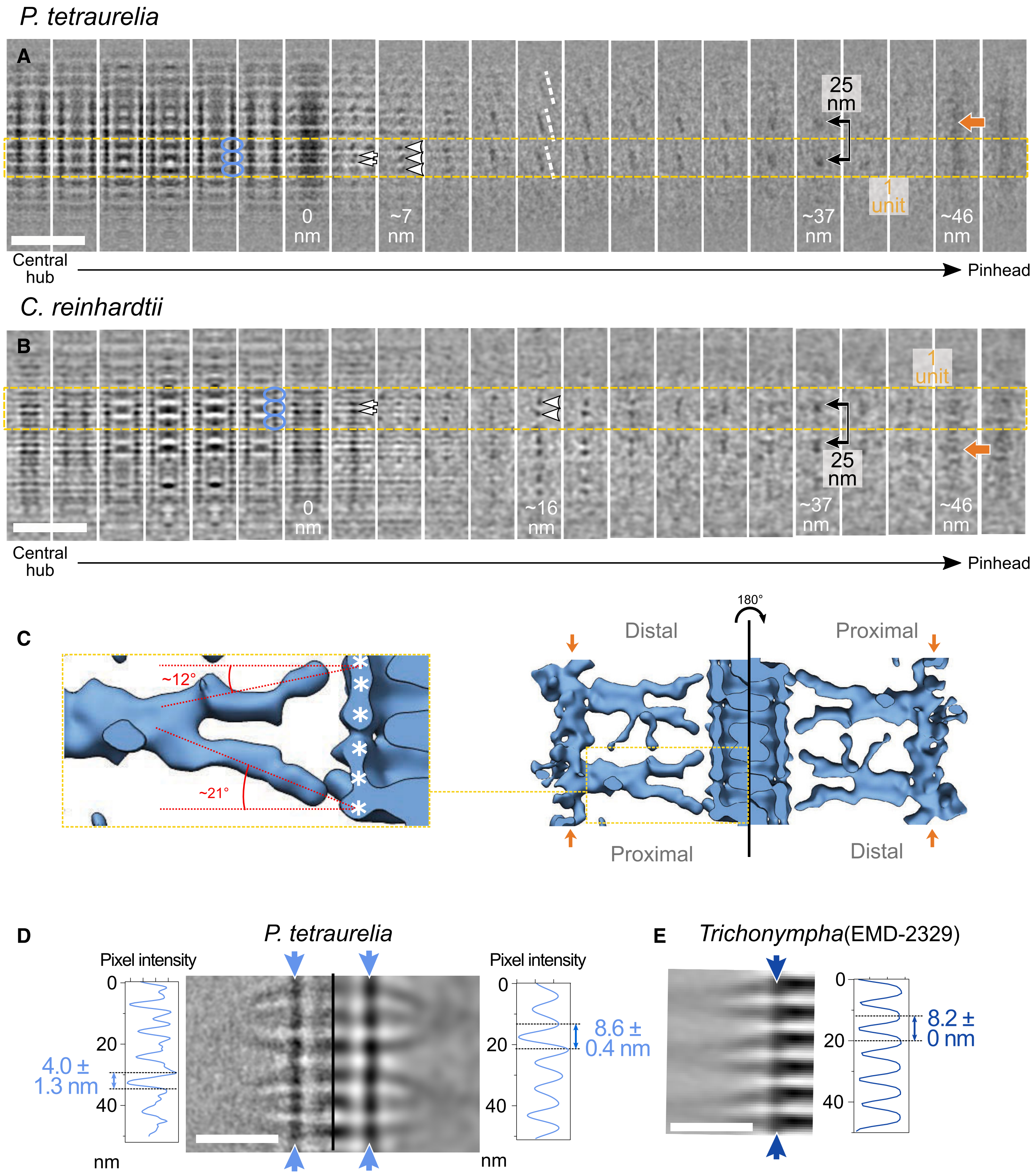

Figure EV4.

 © 2020 The Authors

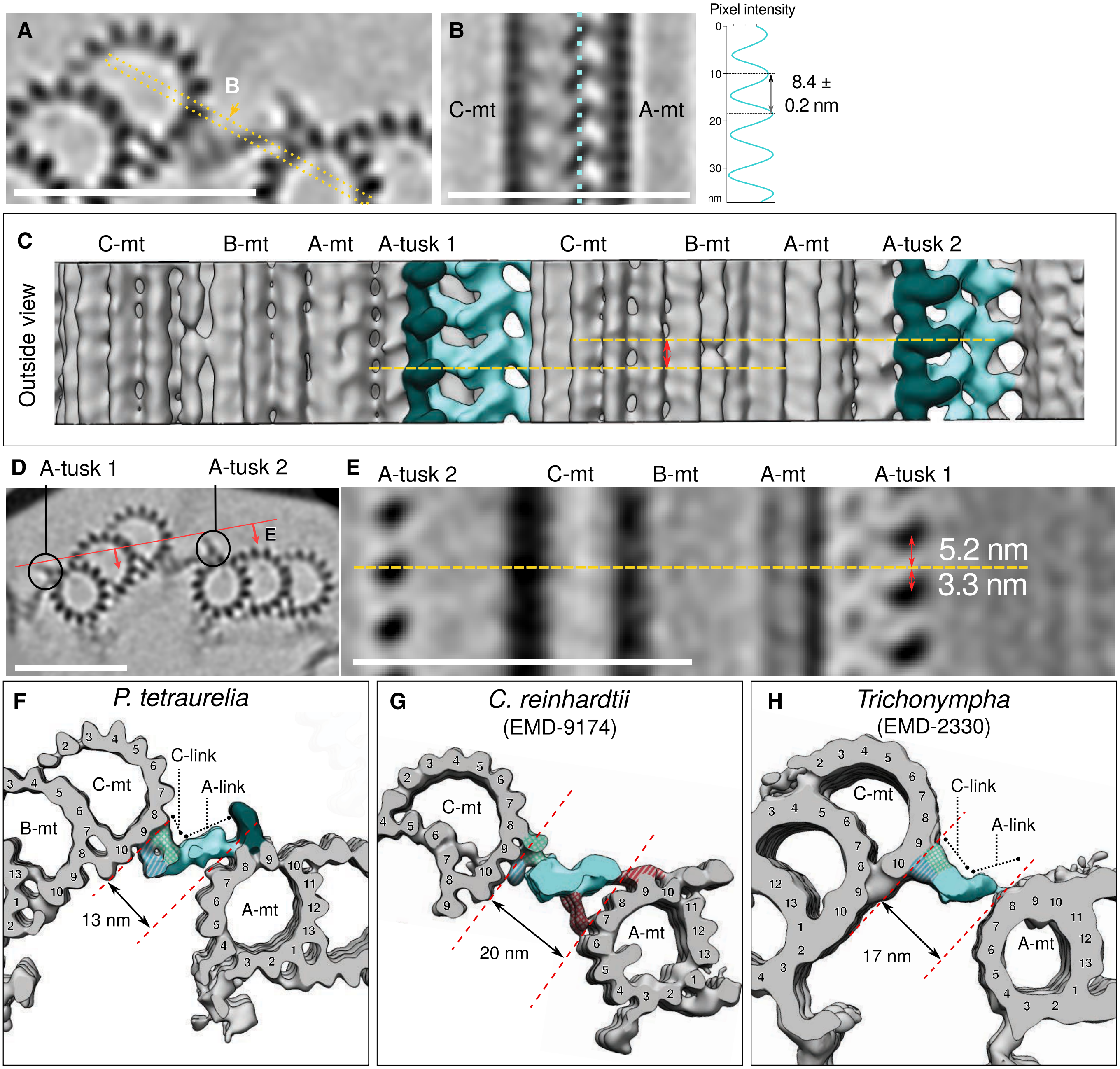

Figure EV5. Architectural features of the *P. tetraurelia* proximal region, and evolutionary comparison of the A-C linker.

A Z-projection of the reconstructed junction between adjacent proximal microtubule triplets. The yellow dotted line and arrow indicate the position and direction of the reslice. Scale bar, 50 nm.

B Cross section highlighting the lateral periodicity of the A-C linker's trunk and its associated plot profile (right) measured along the light blue dotted line. Scale bar, 50 nm.

C Three-dimensional view of two adjacent proximal microtubule triplets seen from the outside of the centriole. Yellow dashed lines indicate the position of the A-tusk from the adjacent triplet. The double-headed red arrow indicates the longitudinal shift between the position of two consecutive A-tusks along the centriole's long axis.

D Projection image of two adjacent proximal microtubule triplets. The red line indicates the position of the cross section shown in (E). Scale bar, 50 nm.

E Cross section of two proximal microtubules triplets showing the longitudinal shift of the A-tusk on one triplet (A-tusk 2) compared to the A-tusk on the adjacent triplet (A-tusk 1). Horizontal line along two consecutive MTTs crossing an A-tusk 2. The red arrows indicate the distances between the A-tusk 2 position along z with the A-tusks 1 (one upper, one lower) from the following MTT. Scale bar, 50 nm.

F–H Three-dimensional views of *P. tetraurelia* (F), *C. reinhardtii* (G, EMD-9174, filtered to 45 Å), and *Trichonympha* (H, EMD-2330). The dotted red lines define the distance between consecutive microtubule triplets. Note that this distance varies between species. Microtubule triplets are in gray, and the A-C linker is in light blue/green. Dashed blue: arms A and B, blue: trunk, red: legs. Dark green: A-tusk.

© 2020 The Authors
