# Supplementary material for: Architecture of the centriole cartwheel‐containing region revealed by cryo‐electron tomography
Source: EMBO J. 2020 Sep 20;39(22):e106246. doi: 10.15252/embj.2020106246 (PMC7667884; doi:10.15252/embj.2020106246)
Supplement: Supplementary file 3 — Movie EV1 [file EMBJ-39-e106246-s003.zip › Movie EV1 legend.docx]

**Movie EV1. 3D rendering of *P. tetraurelia* cartwheel**

The central hub as well as the spokes of *P. tetraurelia* cartwheel are displayed. Note that the spokes are tilted.
